# Oral Immunization With a Plant HSP90-SAG1 Fusion Protein Produced in Tobacco Elicits Strong Immune Responses and Reduces Cyst Number and Clinical Signs of Toxoplasmosis in Mice

**DOI:** 10.3389/fpls.2021.726910

**Published:** 2021-10-04

**Authors:** Edwin F. Sánchez-López, Mariana G. Corigliano, Sonia Oliferuk, Victor A. Ramos-Duarte, Maximiliano Rivera, Luisa F. Mendoza-Morales, Sergio O. Angel, Valeria A. Sander, Marina Clemente

**Affiliations:** ^1^Laboratorio de Molecular Farming y Vacunas, Instituto Tecnológico Chascomús (INTECH), Universidad Nacional de General San Martín (UNSAM), Consejo Nacional de Investigaciones Científicas y Técnicas (CONICET), Chascomús, Argentina; ^2^Laboratorio de Parasitología Molecular, Instituto Tecnológico Chascomús (INTECH), Universidad Nacional de General San Martín (UNSAM), Consejo Nacional de Investigaciones Científicas y Técnicas (CONICET), Chascomús, Argentina

**Keywords:** heat shock protein, SAG1, fusion protein, adjuvant, vaccine, toxoplasmosis, *Nicotiana benthamiana*

## Abstract

Plant 90kDa heat shock protein (HSP90) is a potent adjuvant that increases both humoral and cellular immune responses to diverse proteins and peptides. In this study, we explored whether *Arabidopsis thaliana* HSP90 (AtHsp81.2) can improve the immune effects of a *Toxoplasma gondii* surface antigen 1 (SAG1). We designed two constructs containing the sequence of mature antigen (SAG1_m_), from aa_77_ to aa_322,_ and B- and T-cell antigenic epitope-containing SAG1_HC_, from aa_221_ to aa_319_ fused to AtHsp81.2 sequence. When comparing the transient expression in *Nicotiana tabacum* X-27-8 leaves, which overexpress the suppressor helper component protease HC-Pro-tobacco etch virus (TEV), to that in *N. benthamiana* leaves, co-agroinfiltrated with the suppressor p19, optimal conditions included 6-week-old *N. benthamiana* plants, 7-day time to harvest, *Agrobacterium tumefaciens* cultures with an OD_600nm_ of 0.6 for binary vectors and LED lights. While AtHsp81.2-SAG1_m_ fusion protein was undetectable by Western blot in any of the evaluated conditions, AtHsp81.2–SAG1_HC_ was expressed as intact fusion protein, yielding up to 90μg/g of fresh weight. Besides, the AtHsp81.2–SAG1_HC_ mRNA was strongly expressed compared to the endogenous *Nicotiana tabacum* elongation factor-alpha (NtEFα) gene, whereas the AtHsp81.2–SAG1_m_ mRNA was almost undetectable. Finally, mice were orally immunized with AtHsp81.2–SAG1_HC_-infiltrated fresh leaves (plAtHsp81.2–SAG1_HC_ group), recombinant AtHsp81.2–SAG1_HC_ purified from infiltrated leaves (rAtHsp81.2–SAG1_HC_ group), non-infiltrated fresh leaves (control group), or phosphate-buffered saline (PBS group). Serum samples from plAtHsp81.2–SAG1_HC_-immunized mice had significantly higher levels of IgGt, IgG2a, and IgG2b anti-SAG1_HC_ antibodies than serum from rAtHsp81.2–SAG1_HC_, control, and PBS groups. The number of cysts per brain in the plAtHsp81.2–SAG1_HC_-immunized mice was significantly reduced, and the parasite load in brain tissue was also lower in this group compared with the remaining groups. In an immunoblot assay, plant-expressed AtHsp81.2-SAG1_HC_ was shown to react with antibodies present in sera from *T. gondii*-infected people. Therefore, the plant expression of a *T. gondii* antigen fused to the non-pathogenic adjuvant and carrier plant HSP90 as formulations against *T. gondii* can improve the vaccine efficacy, and plant extract can be directly used for vaccination without the need to purify the protein, making this platform a suitable and powerful biotechnological system for immunogenic antigen expression against toxoplasmosis.

## Introduction

Toxoplasmosis is caused by the parasite *Toxoplasma gondii*, an opportunistic pathogen that infects humans and warm-blooded animals (Peng et al., 2011). In humans, *T. gondii* is an efficient parasite, affecting one in three people (Pittman and Knoll, 2015). Those with the highest risk of developing serious infections are pregnant women, children with congenital infections, and immunocompromised patients (Petersen et al., 2010). Congenital toxoplasmosis is the second most frequent intrauterine parasitic infection (Bojar and Szymańska, 2010); therefore, regular monitoring of pregnant women for this infection is mandatory (Oz, 2014). Furthermore, this parasite is geographically widely spread, and chronically infected animals are a permanent source of parasite transmission for humans (Kijlstra and Jongert, 2008; Tenter, 2009; Sander et al., 2018). In fact, the Center for Disease Control (CDC) in the United States includes *T. gondii* in a list of neglected parasitic infections.^1^ While the cat is the definitive host, mammals and birds are intermediate hosts. *T. gondii* infection in livestock is an important source of economic losses owing to resultant spontaneous abortions and neonatal deaths, especially in sheep and goats (Dubey et al., 1998). The European Union has estimated that economic losses owing to abortions in these species are between 0.7 and 1.4 million euros per year (Katzer et al., 2011). For all these reasons, prevention of toxoplasmosis is a priority including through the development of effective vaccines (Pittman and Knoll, 2015).

Protein-based subunits vaccines are generally considered safe; however, they can be weakly immunogenic, often requiring the addition of adjuvants to elicit an effective immune response (Perez et al., 2012; Jo et al., 2021). Despite several adjuvants have been assayed in experimental subunit vaccines against *T. gondii* (Sander et al., 2018; Şahar et al., 2020), the search for safer and non-toxic adjuvants capable of strongly boosting and directing immune responses is still one of the main challenges in this field. Several plant molecules have shown adjuvant capacity, including saponins, polysaccharides, and lectins (Sander et al., 2018). In addition, in the last decade, heat shock proteins (HSPs) have emerged as new potent plant adjuvants (Buriani et al., 2011, 2012; Corigliano et al., 2013; Bengoa-Luoni et al., 2019; Sánchez-López et al., 2019). HSP90s have been proposed as danger signals since they are inducible proteins whose synthesis increases under certain stress situations such as heat, cold, irradiation, or presence of some pathogens such as viruses and bacteria, and even bacterial toxins (Moseley, 1998). Therefore, when HSP90s are released into the extracellular environment due to cell damage or necrosis, they can bind to surface receptors present on professional antigen-presenting cells (pAPCs) and trigger a pro-inflammatory immune response, thereby modulating the innate immune response (Binder et al., 2000). In particular, this ability occurs, at least, through interaction with TLR2 and TLR4 receptors (Rico et al., 2002). Since both foreign and endogenous HSP90s are involved in the presentation of antigens to pAPCs and in the maturation of these cells, the potential role of HSP90s as adjuvants was recognized favoring antigen presentation (Suzue and Young, 1996; More et al., 1999; Basu et al., 2001; Echeverria et al., 2006; Tobian et al., 2014; Corigliano et al., 2021). Corigliano et al. (2011) showed that plant cytosolic HSP90s, *Nicotiana benthamiana* Hsp90.3 (NbHsp90.3) and *Arabidopsis thaliana* Hsp81.2 (AtHsp81.2), stimulate the proliferation of B cells and that TLR4 is involved in this mitogenic interaction. In fact, TLR4 plays a very important role in immunity against *T. gondii*, but also in its pathogenicity; therefore, its moderate stimulation is sought in a vaccine against toxoplasmosis (Zare-Bidaki et al., 2014).

Several vaccine candidates of *T. gondii*, including membrane surface antigens and secretory antigens, have been tested either individually or as multi-antigen vaccines (Clemente, 2014; Sander et al., 2018; Loh et al., 2019). Surface antigen 1 (SAG1) is the main surface antigen from *T. gondii*, and it was the first vaccine antigen of interest from *T. gondii* to be expressed in plants (Clemente et al., 2005; Laguía-Becher et al., 2010). SAG1 contains a signal peptide required to entry into the endoplasmic reticulum and then is cleaved at its C-terminal hydrophobic region, to generate a SAG1 mature version (SAG1_77-322_), being modified with a glycosylphosphatidylinositol anchor that is important to *T. gondii* surface localization (Chen et al., 2001). Besides, its amino acid sequence presents 12 cysteine residues involved in the formation of disulfide bridges and correction of protein folding (Burg et al., 1988). Recently, Sánchez-López et al. (2019) produced a fusion protein in bacteria with the C-terminal region of SAG1_221-319_, containing B- and T-cell antigenic epitopes (Godard et al., 1994; Siachoque et al., 2006; Cardona et al., 2009), fused to NbHsp90.3 as an adjuvant (NbHsp90.3-SAG1_HC_). They observed that NbHsp90.3-SAG1_HC_ presented a more potent protective immune response through a biased-Th1 type cytokine production than mature version of SAG1 (SAG1_m_) alone (Corigliano et al., 2011; Bengoa-Luoni et al., 2019). On the other hand, the mixture of AtHsp81.2+*Neospora caninum* SAG1 (Bengoa-Luoni et al., 2019) increased humoral and cellular immune responses against these antigens, evoking the secretion of high interferon (IFN)-γ levels and inducing protection in mouse models. It is important to note that the fusion of the antigen of interest to HSP90 generates a Th1-biased immune response (Echeverria et al., 2006; Corigliano et al., 2013; Pishraft-Sabet et al., 2015; Sánchez-López et al., 2019), whereas the mixture of HSP90 and the evaluated antigen does not always generate this immune profile (Zhu et al., 2016; Chung et al., 2017; Bengoa-Luoni et al., 2019). Therefore, we consider that plant HSP90 is effective as adjuvants when it is incorporated as fusion protein in the design of novel vaccines to prevent infectious diseases, including toxoplasmosis.

Most of the recombinant molecules currently available for medical use are synthesized in microbial and mammalian cell cultures (Fischer et al., 2014). Although cell lines allow the synthesis of eukaryotic proteins in a similar system to that of their origin, there is a high initial economic cost and great investments scale for protein production due to the cost-intensive bioreactor-based infrastructure (Paul et al., 2013). In addition, these systems carry serious economic risks in case of contamination in the production line, in many cases also being unsafe for human health (Fischer et al., 2013). The demand for therapeutic recombinant proteins and peptides for medical and veterinary use has stimulated the development of novel expression systems that do not carry the immanent risk of contamination with human viruses, prions or bacterial endotoxin, simpler and more cost-effective technologies, which allow large-scale protein production (Paul et al., 2013). One alternative method for this is the production of recombinant proteins in plants, called plant molecular farming (Tschofen et al., 2016). Over the years, several studies have shown that this technology is a viable commercial alternative method of production (Paul et al., 2015; Yao et al., 2015). However, this method often produces from 20 to 100mg of recombinant proteins per kg of plant biomass, which limits the use of plant-based production platforms (Doran, 2006; Streatfield, 2007; Holtz et al., 2015; Menzel et al., 2016). Several strategies to reduce proteolytic activity have been developed and tested in a variety of plant-based expression systems with the aim of increasing the accumulation levels higher than 2g of recombinant therapeutic proteins per kg of plant biomass (Hehle et al., 2011; Castilho et al., 2014; Holtz et al., 2015; Mandal et al., 2016; Tschofen et al., 2016; Yamamoto et al., 2018; Buyel, 2019; Kopertekh and Schiemann, 2019; Peyret et al., 2019; Diamos et al., 2020). The use of silencing suppressors is also another approach to boost the accumulation of recombinant proteins in plants (Voinnet et al., 2003; Dhillon et al., 2009; Bashandy et al., 2015; Matsuo et al., 2016; Norkunas et al., 2018). However, more research is needed so that plant molecular farming becomes competitive with microbial and mammalian cells that yield typically 5–10g of recombinant protein per liter of culture which correspond to 5–10g of recombinant protein per kg of plant biomass (Kaufman and Kalaitzandonakes, 2011; Santos et al., 2016; Kelley, 2020). Identification of new strategies for minimizing the degradation of foreign proteins would improve the potential for commercialization of plant-based systems that produce proteins of industrial or pharmaceutical interest. In particular, SAG1 is a good candidate to optimize production using alternative expression systems including those based in plants, since its accumulation is difficult to elicit using traditional systems as bacteria, yeast, or mammals cells (Kim et al., 1994; Harning et al., 1996; Chen et al., 2001; Letourneur et al., 2001; Meek et al., 2003; Nigro et al., 2003). *Escherichia coli*-based expression systems showed to be inadequate for the SAG1 production, obtaining a misfolded, insoluble, and poorly immunogenic protein (Harning et al., 1996; Nigro et al., 2003), while the SAG1 expression using eukaryotic cell systems, such as *Pichia pastoris* (Letourneur et al., 2001) or Chinese hamster ovary (CHO) cells (Kim et al., 1994), resulted in a hyper-glycosylation of the protein (N- and O-glycosylation) that affected its immunogenicity.

In this study, we explored the transient expression in plant of SAG1_m_ and SAG1_HC_ fused to the adjuvant/carrier AtHsp81.2 in plants. This strategy could drive down vaccine costs and simplify production, as both antigen and adjuvant are produced in the same plant (Kapila et al., 1997; Laguía-Becher et al., 2010). Vacuum and syringe agroinfiltration methods were carried out on *N. tabacum* X-27-8 leaves, which overexpress the post-transcriptional gene silencing suppressor helper component protease (HC-Pro)-*tobacco etch virus* (TEV) or on *N. benthamiana* leaves co-agroinfiltrated with the suppressor p19 from the *tomato bushy stunt virus* (TBSV). An additional advantage of this system is that AtHsp81.2 may act as a plant host chaperone to stabilize and improve the accumulation of the protein of interest, in this case SAG1.

## Materials and Methods

### Construction of Expression Vectors in Plants

The sequence of SAG1_m_ from aa_77_ to aa_322_ (Laguía-Becher et al., 2010) and SAG1_HC_ from aa_221_ to aa_319_ (Sánchez-López et al., 2019) was amplified from the pRSETA–SAG1_77-322_ vector (Cóceres et al., 2010) using PCR. These sequences do not contain the N-terminal signal peptide or the C-terminal hydrophobic region (Supplementary Figure S1). The truncated version of the protein, SAG1_HC_, contained T- and B-cell epitopes (Godard et al., 1994; Siachoque et al., 2006; Cardona et al., 2009).

**Figure 1:**
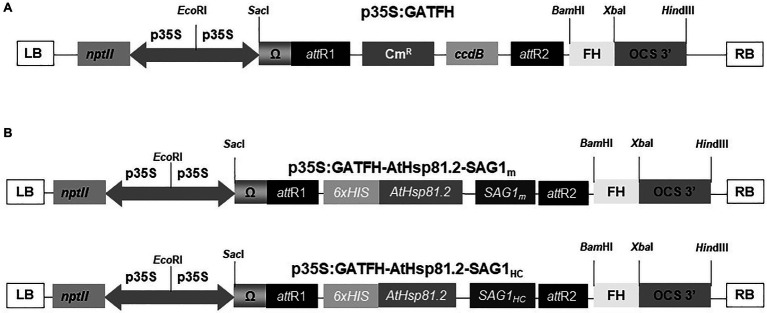
Schematic representation of constructs used in the *Agrobacterium tumefaciens*-mediated transient expression systems. **(A)** Binary plant vector p35S:GATFH; **(B)** AtHsp81.2–SAG1_m_ and AtHsp81.2–SAG1_HC_ constructs. p35S, cauliflower mosaic virus (CaMV) 35S promoter; T-OCS, octopine synthase terminator sequence; Ω, translational enhancer sequence; nptII, kanamycin resistance gene; attR1 and attR2, sites of recombination; FH, fumarate hydratase sequence; *Cm^R^*, chloramphenicol resistance; *ccdB*, toxin ccdB protein for the *E. coli* strains; 6x His, six-histidine tag; RB and LB, right and left borders of the T region, respectively.

For SAG1_m_, the forward primer sequence was 5′ ggt acc AT TTC ACT CTC AAG TGC 3′ and the reverse primer sequence was 5′ aag ctt CTA AAT GGA AAC GTG ACT GGC 3′; for SAG1_HC_, the forward primer sequence was 5′ ggt acc AT AAA GTT CCT CAA GAC AAC 3′ and the reverse primer sequence was 5′ aag ctt CTA AAT GGA AAC GTG ACT GGC 3′. For both sequences, primers were flanked by *KpnI* and *HindIII* restriction sites (shown in the sequences as lowercase letters), respectively, at the 5′ and 3′ ends. The amplified SAG1_m_ and SAG1_HC_ sequences were cloned into the pRSETA–AtHsp81.2 vector (Corigliano et al., 2011) in-frame with the AtHsp81.2 sequence. AtHsp81.2–SAG1_m_ and AtHsp81.2–SAG1_HC_ sequences were amplified including the 6x histidine (His) tag present in the pRSETA vector using the forward primer sequence 5′ ATG CGG GGT TCT CAT CAT CAT CAT CAT CAT 3′ for both AtHsp81.2–SAG1_m_ and AtHsp81.2–SAG1_HC_ and the reverse primer sequences 5′ aag ctt CTA AAT GGA AAC GTG ACT GGC- 3′ or 5′ aag ctt CTA AAT GGA AAC GTG ACT GGC- 3′, respectively. The resulting fragments were cloned into the pCR8TM/GW/TOPO^®^ TA vector (Invitrogen, Carlsbad, CA, United States). After sequencing, the recombinant plasmids were used to subclone into the binary plant vector p35S:GATFH using Gateway^™^ LRClonase^™^ II enzyme mix according to the manufacturer’s protocol (Invitrogen; Figure 1A). AtHsp81.2–SAG1_m_ and AtHsp81.2–SAG1_HC_ genes were inserted between the Cauliflower Mosaic Virus (CaMV) 35S promoter (p35S) and octopine synthetase terminator (t-OCS; Figure 1B).

### Agroinfiltration

*Agrobacterium tumefaciens* (recently renamed to *Rhizobium radiobacter*) strain GV3101 (Rif^R^/Gm^R^) bacteria were separately transformed with the constructs p35S:GATFH–AtHsp81.2–SAG1_m_ and p35S:GATFH–AtHsp81.2–SAG1_HC_, using the freeze–thaw method (Hofgen and Willmitzer, 1988). Cultures were grown with shaking at 28°C in lysogeny broth (LB) medium containing kanamycin (50mg/L). *A. tumefaciens* cultures were then diluted in LB medium to an OD_600nm_ of 0.1, 0.6, or 1.0 and used to agroinfiltrate *Nicotiana benthamiana* or *N. tabacum* X-27-8 leaves (Anandalakshmi et al., 1998). *Nicotiana benthamiana* was also co-agroinfiltrated with the recombinant *A. tumefaciens* carrying the constructs and a plasmid encoding the p19 protein from TBSV, a suppressor of gene silencing (Voinnet et al., 2003) at OD_600nm_ of 0.6. The *N. tabacum* X-27-8 plants express high levels of the HC-Pro-TEV sequence (Brigneti et al., 1998). Leaves from 6-week old (without flower buds) or 8-week old (with flower buds) were infiltrated using either vacuum or syringe infiltration methods (Kapila et al., 1997; Laguía-Becher et al., 2010). Briefly, vacuum agroinfiltration was performed by using vacuum pump. Entire plants were submerged into bacterial suspensions and introduced into a desiccator connected to a vacuum pump that exerted pressure between 500 and 600mmHg, allowing the *Agrobacterium* culture to get into the leaves. Furthermore, syringe agroinfiltration was performed by using syringes without needles. The bacterial solution was forced to enter into entire leaves by gently pushing the piston using a uniform pressure during tissue infiltration. The bacterial suspension was inoculated into the air spaces within the leaf through the stomata. Then, whole plants were incubated for 3–7days under controlled temperature and lighting conditions in chambers with either fluorescent or LED lights (23°C/16h photoperiod/photon flux density 200μmolms^-1^; Albarracín et al., 2015).

### RNA Isolation and Gene Expression Analysis

Leaf samples from three *N. benthamiana* plants independently agroinfiltrated with different *A. tumefaciens* cultures containing AtHsp81.2–SAG1_HC_ and p19 or AtHsp81.2–SAG1_m_ and p19 were collected at 2, 4, and 7days post-agroinfiltration. Briefly, 100μg of leaves was ground in liquid nitrogen for RNA extraction. Total RNA was extracted using the TRIzol reagent (Invitrogen) following manufacturer’s instructions and treated with TURBO DNase-Free Kit (Ambion, Naugatuck, CT, United States). RNA concentration and integrity were analyzed as described previously (Corigliano et al., 2011). cDNA synthesis was performed using M-MLV reverse transcriptase (Promega, Madison, WI, United States) and 2μg of total RNA as a template from each sample. Subsequently, a 4-fold dilution of the cDNA was used as the RT-qPCR reaction templates (SYBR Green Master Mix, Invitrogen). A portion of 300 base pairs of the *SAG1* gene encoding B- and T-cell epitopes was amplified using the forward primer 5′ GGT ACC AAA GTT CCT CAA GAC AAC AAT CAG 3′ and reverse primer 5′ AAG GTT CTA AAT GGA AAC GTG ACT GGC TGT TCC C 3′. Its expression was quantified relative to the *N. tabacum* elongation factor-alpha (*NtEFα*) gene, used as an endogenous control (forward primer 5′ TGA GAT GCA CCA CGA AGC TC 3′ and reverse primer 5′ CCA ACA TTG TCA CCA GGA AGT G 3′). Relative quantification was achieved using the comparative cycle threshold method. The amplification reactions were performed using a StepOnePlus^™^ real-time PCR system (Applied Biosystems, Foster City, CA, United States).

### Protein Extraction

Leaves from plants agroinfiltrated with recombinant *A. tumefaciens* and leaves from non-agroinfiltrated plants were ground separately in liquid nitrogen. For total protein extraction in each sample, 100mg of ground leaves was homogenized in 300μl Laemmli buffer (0.5M Tris–HCl pH 6.5, 4% sodium dodecyl sulfate (SDS), 20% glycerol, 10% β-mercaptoethanol, and 0.1% bromophenol blue). The total protein content was measured using the reducing agent and detergent compatible (RC DC) protein assay (Bio-Rad, Hercules, CA, United States) with bovine serum albumin (BSA) as a standard according to the manufacturer’s instructions.

### Purification of Plant-Expressed AtHsp81.2–SAG1_HC_

Leaves from agroinfiltrated plants with recombinant *A. tumefaciens* and leaves from non-agroinfiltrated plants were ground in liquid nitrogen, suspended in extraction buffer [250mm sucrose, 50mm N-2-hydroxyethylpiperazine-N-ethanesulfonic acid (HEPES) buffer pH 8.0, 1 mm ethylenediaminetetraacetic acid (EDTA), 20mm β-mercaptoethanol, 0.06% L-cysteine, 1mm MgCl_2_, 0.6% polyvinylpyrrolidone (PVP), 2% triton, and 1% phenylmethylsulfonyl fluoride (PMSF)], and incubated at 4°C overnight. After centrifugation (10,000g), the supernatants containing the total soluble proteins (TSP) were loaded in a nitrilotriacetic-acid-Ni^2+^ column (Ni-NTA; Qiagen, Germantown, MD, United States). The proteins that were retained through the column were washed with wash buffer (PBS pH 8.0, 10mm imidazole, 1% PMSF), and AtHsp81.2-SAG1_HC_ was eluted with elution buffer (PBS pH 8.0, 250mm imidazole, 1% PMSF). AtHsp81.2–SAG1_HC_ was purified by SDS-polyacrylamide gel electrophoresis (10% SDS-PAGE) using the Mini-Protean system III (Bio-Rad) and was either stained with Coomassie brilliant blue or transferred onto polyvinylidene fluoride (PVDF) membranes (GE Healthcare, Wauwatosa, WI, United States) using an electro-transfer unit (Bio-Rad).

### SDS-PAGE and Western Blot Analysis

Leaf protein extracts and purified AtHsp81.2–SAG1_HC_ (rAtHsp81.2–SAG1_HC_) were heated at 100°C for 5min, separated by 10% SDS-PAGE gels, and transferred onto PVDF membrane (GE Healthcare) using an electro-transfer unit (Bio-Rad). After blocking, the membranes were incubated with mouse anti-*E. coli*-purified SAG1_m_ (rEcSAG1_m_ polyclonal antibody (1:500; Laguía-Becher et al., 2010) followed by anti-mouse IgG alkaline phosphatase conjugate as the secondary antibody (1:5,000; Sigma-Aldrich, St. Louis, MO, United States). Phosphatase activity was detected using p-nitroblue tetrazolium chloride (NBT)/5-bromo-4-chloro-3-indolyl phosphate (BCIP) substrate (Promega). The levels of AtHsp81.2–SAG1_m_ and AtHsp81.2–SAG1_HC_ in leaves and rAtHsp81.2–SAG1_HC_ were estimated using a serial dilution of a known concentration of rEcSAG1_m_ (Cóceres et al., 2010; Laguía-Becher et al., 2010), electrophoresed on the same polyacrylamide gel. The band intensity of rEcSAG1_m_ detected by Western blot was estimated using the Gel-Pro analyzer software (Media Cybernetics, Rockville, MD, United States) and used as a standard to build a calibration curve as previously described (Laguía-Becher et al., 2010; Yácono et al., 2012). The leaf-expressed band intensity detected by Western blot was also estimated using the Gel-Pro analyzer software and compared with the calibration curve. This analysis allowed us to estimate the concentration of proteins expressed by the agroinfiltrated plants.

### Mouse Models

C57BL/6 (H-2b)-specific pathogen-free mice were obtained from the central bioterium of Facultad de Ciencias Exactas y Naturales of Universidad de Buenos Aires. Six- to eight-week-old female mice were bred and housed following the institutional guidelines of the Universidad de General San Martín (C.I.C.U.A.E., IIB-UNSAM, 09/2016). Mice had access to food and water *ad libitum* and were maintained at 21–22°C and a 12:12-h light:dark photocycle.

### Oral Immunization of Mice

In all cases, eight mice per experimental group were deprived of food and water for 2h prior to each immunization. One group was gavaged with fresh pulverized leaf extracts containing the equivalent of approximately 1μg (first–fourth doses) or 2μg (fifth dose) of AtHsp81.2-SAG1_HC_ and homogenized in 200μl of PBS 1x buffer at 7-day intervals (plAtHsp81.2-SAG1_HC_ group). A second group received approximately 1μg (first–fourth doses) or 2μg (fifth dose) of rAtHsp81.2–SAG1_HC_ purified from infiltrated leaves at 7-day intervals (rAtHsp81.2-SAG1_HC_ group). The control group received five doses of non-infiltrated leaf extracts homogenized in 200μl of PBS 1x buffer, while the PBS control group received 5 doses of PBS 1x buffer (PBS group).

### Assessment of Antibody Responses

Before each immunization, blood was collected from the cheek, and serum was stored at 20°C until analysis for the presence of specific antibodies. Pre-immunization serum samples were taken for using as negative controls. Antigen-specific antibodies were analyzed by ELISA according to a previously described protocol (Clemente et al., 2005; Laguía-Becher et al., 2010). Briefly, each well of a 96-well microtiter plate (Immuno Plate Maxisorp; Nunc, Rochester, NY, United States) was coated overnight at 4°C with 5μg/ml of rEcSAG1_m_ or rEcAtHsp81.2 diluted in 50mm carbonate buffer (pH 9.6). Rat anti-mouse immunoglobulin G (IgG)-horseradish peroxidase conjugate (1:3,000) was used as a secondary antibody (Sigma-Aldrich), and rat anti-mouse IgG1, IgG2a, or IgG2b horseradish peroxidase conjugates (1:10,000; Sigma-Aldrich) were used for isotype analysis. Immune complexes were revealed using tetramethylbenzidine substrate (TMB, One-Step; Invitrogen) incubated for 30min at room temperature in the dark, before adding 50μl of 2M H_2_SO_4_ to stop the reaction and measuring the OD_450nm_ with a *λ* correction of 570nm using an automatic ELISA reader (Synergy H1; Bio-Tek, Winooski, VT, United States). Serum samples were diluted 1:100 and 1:12,800. To attain a comparative analysis of IgG profiles, serum samples were used at a 1:100 dilution.

### Experimental Infection With *Toxoplasma gondii* Cysts

Mice (eight per experimental group) were orally infected with 20 *T. gondii* Me49 tissue cysts (a sublethal dose) 2weeks after the last immunization was given (Yácono et al., 2012; Albarracín et al., 2015). Me49 tissue cysts were isolated from brain of experimentally infected mice. Mice were observed daily to evaluate clinical signs of toxoplasmosis and mortality rate. One month after this challenge, mice were sacrificed, and their brains were removed. Each brain was homogenized in 2ml of PBS with a Dounce tissue grinder. The mean number of cysts per brain was determined by counting under an optical microscope; four samples of 20μl aliquots were counted for each homogenized brain.

### Quantification of Parasite Load Using qPCR

Extraction of genomic DNA was performed using 7×10^7^ tachyzoites (M49 strain) and 0.05mg of mouse brain tissue using the Accuprep Genomic DNA extraction kit (Bioneer, Daejeon, Korea) following the manufacturer’s conditions. The parasite load in the mouse brain tissue was quantified as previously described by Pinitkiatisakul et al. (2008). Briefly, quantitative PCR (qPCR; SYBR Green Master Mix, Invitrogen) was performed using a StepOnePlus qPCR system (Applied Biosystems, Foster City, CA, United States). *Toxoplasma gondii* DNA in the samples of mouse brain tissue was detected using the forward primer 5′ CAC AGA AGG GAC AGA AGT 3′ and reverse primer 5′ TCG CCT TCA TCT ACA GTC 3′, designed to amplify 529bp repetitive sequence (Edvinsson et al., 2006; accession numbers AF146527 and AF487550). Results were expressed as ng of parasite DNA per mg brain tissue using absolute quantification using a serial dilution curve of *T. gondii* DNA in a range of 0.069–6,900pg. Amplification of a 71bp fragment from the rRNA S28 from *Mus musculus* (GenBank accession no. X00525) using the forward primer 5′ TGC CAT GGT AAT CCT GCT CA 3′ and reverse primer 5′ CCT CAG CCA AGC ACA TAC ACC 3′ as an internal control.

### Reactivity of Human Serum Samples

Human serum samples were obtained from the Centro de Toxoplasmosis of the Hospital Alemán, a referral center for toxoplasmosis in Argentina. Samples were analyzed using the Sabin–Feldman serological dye test for toxoplasmosis and IgG and IgM indirect immunofluorescence (IIF; Nigro et al., 2003). Serum samples were classified as positive (with either an acute or chronic infection) or negative. The use of these serum samples was approved by the Independent Ethics Committee of the Medical Federation of Buenos Aires Province, Argentina (FE.ME.BA., note 592, September 23, 2014). Leaf extract samples expressing the AtHsp81.2–SAG1_HC_ were separated by 10% SDS-PAGE under reducing conditions and transferred to a PVDF membrane (GE Healthcare). Human serum samples were analyzed at a dilution of 1:100. Alkaline phosphatase-conjugated anti-human IgG was used as the secondary antibody (1:2,500, Sigma-Aldrich). The reaction was developed by the addition of NBT/BCIP substrate (Promega); PageRuler^™^ prestained protein ladder was used as molecular marker (Fermentas, Waltham, MA, United States) and rEcSAG1_m_ as a positive control.

### Statistical Analysis

Statistical analysis was carried out in Prism 5.0 (GraphPad, San Diego, CA, United States) using one- and two-way ANOVA, where appropriate. Tukey’s multiple comparisons test was used to compare means among groups. *p*<0.05 was used as a cutoff for significance.

## Results

### Transient Expression, Quantification, and Purification of AtHsp81.2–SAG1_m_ and AtHsp81.2–SAG1_HC_ in Tobacco Plants

We successfully designed 2 constructs carrying cDNA sequences, the first encoding *T. gondii* SAG1 from aa_221_ to aa_319_ (SAG1_HC_), which contains B- and T-cell antigenic epitopes (Godard et al., 1994; Siachoque et al., 2006; Cardona et al., 2009) associated with humoral and cellular (HC) immune responses (Sánchez-López et al., 2019), and the second encoding SAG1 from aa_77_ to aa_323_ (SAG1_m_), the mature version of the antigen (Laguía-Becher et al., 2010; Supplementary Figure S1). Each was separately fused to the C-terminus of the cytosolic AtHsp81.2, which included an N-terminal His6-tag. Both of the resultant cytosolic fusion proteins (AtHsp81.2–SAG1_HC_ and AtHsp81.2–SAG1_m_) were cloned separately into gateway-compatible p35S:GATA-FH binary vectors (Figure 1). A comparison of different parameters was performed to optimize the cytosolic recombinant AtHsp81.2-SAG1_HC_ and AtHsp81.2-SAG1_m_ proteins (Table 1).

**Table 1:** Parameters evaluated to optimize the transient expression of AtHsp81.2-SAG1_HC_ and AtHsp81.2-SAG1_m_ proteins in X-27-8 transgenic *N. tabacum* and *N. benthamiana*.

Plant type	Silencing suppressor	Infiltration type	Days post-infiltration (days)	Plant age (weeks)	OD_600nm_	Light type	AtHsp81.2-SAG1_m_	AtHsp81.2-SAG1_HC_ (μg/g FW)
X-27-8	–	Syringe or vacuum	5 or 7	6 or 8	0.1, 0.6 or 1.0	LED or fluorescence	n. d.	n. d.
N. b.	–	Syringe or vacuum	5 or 7	6 or 8	0.1, 0.6 or 1.0	LED or fluorescence	n. d.	n. d.
N. b.	p19	Syringe or vacuum	5	6 or 8	0.1, 0.6 or 1.0	LED or fluorescence	n. d.	n. d.
N. b.	p19	Syringe or vacuum	5 or 7	6 or 8	0.1, 0.6 or 1.0	fluorescence	n. d.	n. d.
N. b.	p19	Syringe	7	6	0.6	LED	n. d	90±30
N. b.	p19	Vacuum	7	6	0.6	LED	n. d	42±10
N. b.	p19	Syringe	7	6	0.1	LED	n. d	18±7.5
N. b.	p19	Syringe	7	6	1.0	LED	n. d	7±2
N. b.	p19	Syringe	7	8	0.6	LED	n. d	5±2

N. b., *Nicotiana benthamiana*; X-27-8, *Nicotiana tabacum* X-27-8; n. d., not detected in any of these conditions.

**Figure 2:**
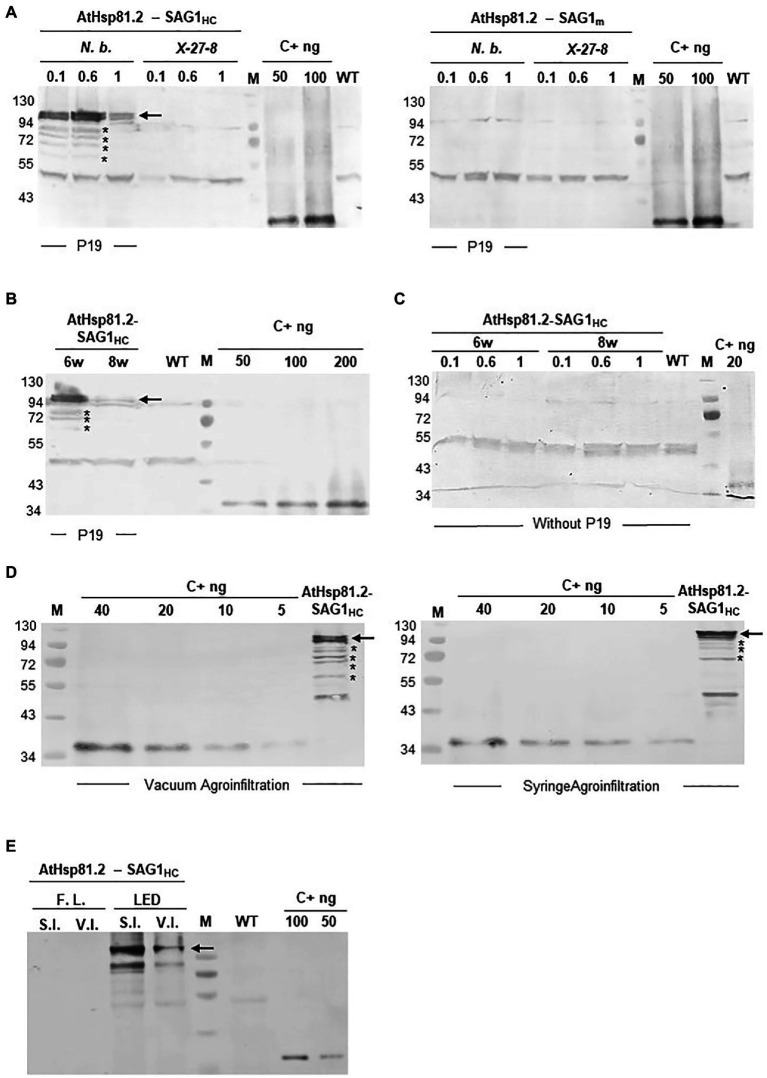
Expression and quantification of AtHsp81.2–SAG1_HC_ and AtHsp81.2–SAG1_m_ produced in *Nicotiana benthamiana* using Western blot analysis. **(A)** Fresh material from *N. benthamiana* leaves or the X-27-8 transgenic line of *N. tabacum* leaves harvested 7days after syringe infiltration using *A. tumefaciens* [optical density (OD)_600nm_=0.1, 0.6 and 1.0]. *Nicotiana benthamiana* leaves were co-agroinfiltrated with the p19 suppressor and AtHsp81.2–SAG1_HC_ or AtHsp81.2–SAG1_m_ constructs, whereas transgenic line X-27-8 leaves were agroinfiltrated with AtHsp81.2–SAG1_HC_ or AtHsp81.2–SAG1_m_ constructs alone. **(B)** Fresh material from *N. benthamiana* leaves of 6- and 8-week-old plants, harvested 7days after syringe infiltration with *A. tumefaciens* (OD_600nm_=0.6). *N. benthamiana* leaves were co-agroinfiltrated with the p19 suppressor and AtHsp81.2–SAG1_HC_. **(C)** Fresh material from *N. benthamiana* leaves of 6- and 8-week-old plants, harvested 7days after syringe infiltration with *A. tumefaciens* (OD_600nm_=0.1, 0.6, and 1.0). *N. benthamiana* leaves were co-agroinfiltrated with AtHsp81.2–SAG1_HC_ without the p19 suppressor. **(D)** Fresh material from *N. benthamiana* leaves of 6-week-old plants, harvested 7 days after syringe or vacuum agroinfiltration with *A. tumefaciens* (OD_600nm_=0.6). *Nicotiana benthamiana* leaves were co-agroinfiltrated with the p19 suppressor and AtHsp81.2–SAG1_HC_. **(E)** Fresh material from *N. benthamiana* leaves of 6-week-old plants and harvested 7days after syringe or vacuum infiltration with *A. tumefaciens* (OD_600nm_=0.6). *N. benthamiana* leaves were co-agroinfiltrated with the p19 suppressor FIGURE 2and AtHsp81.2–SAG1_HC_ and grown under fluorescent or LED lights. Serial dilutions of rEcSAG1_m_ were used as a reference for protein quantification. Lanes were loaded with an equal amount (50μg) of total proteins for direct comparison. WT, agroinfiltrated with *A. tumefaciens* containing an empty expression vector; C+, *E. coli*-purified SAG1_m_ (rEcSAG1_m_); M, prestained molecular weight protein marker (Fermentas).

**Figure 3:**
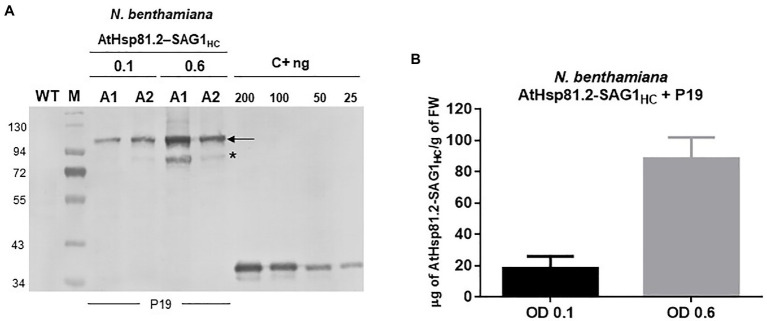
Expression and quantification of plant-produced AtHsp81.2–SAG1_HC_. **(A)** Western blot analysis of fresh material from *Nicotiana benthamiana* leaves from 6-week-old plants harvested 7days after two independent syringe agroinfiltrations (A1 and A2) with *A. tumefaciens* [optical density (OD)_600nm_=0.1 and 0.6, respectively, for A1 and A2]. *N. benthamiana* leaves were co-agroinfiltrated with the p19 suppressor and AtHsp81.2–SAG1_HC_. Serial dilutions of rEcSAG1_m_ were used as reference for protein quantification. Lanes were loaded with equal (50μg) of total proteins for direct comparison. C+, *E. coli*-purified SAG1_m_ (rEcSAG1_m_); M, prestained molecular weight protein marker (Fermentas). **(B)** AtHsp81.2–SAG1_HC_ accumulation in tobacco leaves. The intensity of the band corresponding to SAG1 expressed in *E. coli* and detected by Western blot was used build a quantification calibration curve with standard amounts. Leaf-expressed AtHsp81.2–SAG1_HC_ band intensity in the Western blot was estimated using Gel-Pro analyzer software and compared using the quantification calibration curve.

After agroinfiltration, the optimal conditions for the transient expression of each of the two constructs were established using Western blot with an anti-rEcSAG1m polyclonal antibody (Table 1, Figure 2). Initially, we analyzed the effect of suppressors of gene silencing on protein accumulation levels. Agroinfiltration was carried out either on *N. tabacum* X-27-8 leaves, which overexpress the suppressor HC-Pro- TEV (Brigneti et al., 1998), or on *N. benthamiana* leaves co-agroinfiltrated with the suppressor p19 (Voinnet et al., 2003). AtHsp81.2–SAG1_m_ was not detected in any of these conditions, and AtHSP81.2-SAG_HC_ was not detected in *N. tabacum* X-27-8 leaves (Figure 2A). In contrast, Western blot analysis revealed a 110kDa band as expected for the AtHsp81.2–SAG1_HC_ fusion protein under co-agroinfiltration with the suppressor p19 in *N. benthamiana* leaves (Figure 2A), indicating correct expression. Other bands with lower molecular weights (bands between 90 and 60kDa) were also detected, which likely represent degradation products. Accumulation of AtHsp81.2–SAG1_HC_ in *N. benthamiana* leaves co-agroinfiltrated with suppressor p19 was dependent on the optical density (OD_600nm_) of the construct-containing *Agrobacterium tumefaciens* cultures, plant age, and infiltration type (Table 1 and Figure 2). In 6-week-old *N. benthamiana* plants, syringe infiltration at OD_600nm_ 0.1 and OD_600nm_ 1.0 yielded approximately 18 and 7μg/g of fresh weight (FW), respectively, while an OD_600nm_ 0.6 yielded around 90μg/g of FW (Table 1 and Figure 2A). However, with OD_600nm_ of 0.6, only 5μg/g of FW was obtained in 8-week-old *N. benthamiana* plants (Table 1 and Figure 2B). AtHsp81.2–SAG1_HC_ was not detected when it was infiltrated in *N. benthamiana* without suppressor p19 (Figure 2C). Using vacuum instead of syringe infiltration in 6-week-old plants decreased the yield from 90 to 42μg/g (Table 1 and Figure 2D). Non AtHsp81.2-SAG1_HC_ was detected at 5days post-agroinfiltration or fluorescent lighting conditions (Table 1 and Figure 2E). Therefore, AtHsp81.2-SAG1_HC_ had the highest accumulation under the following conditions: syringe infiltration with suppressor p19, OD_600nm_ 0.6, 6-week-old *N. benthamiana* plants, 7days post-infiltration, and LED lighting (Table 1 and Figure 3).

**Figure 4:**
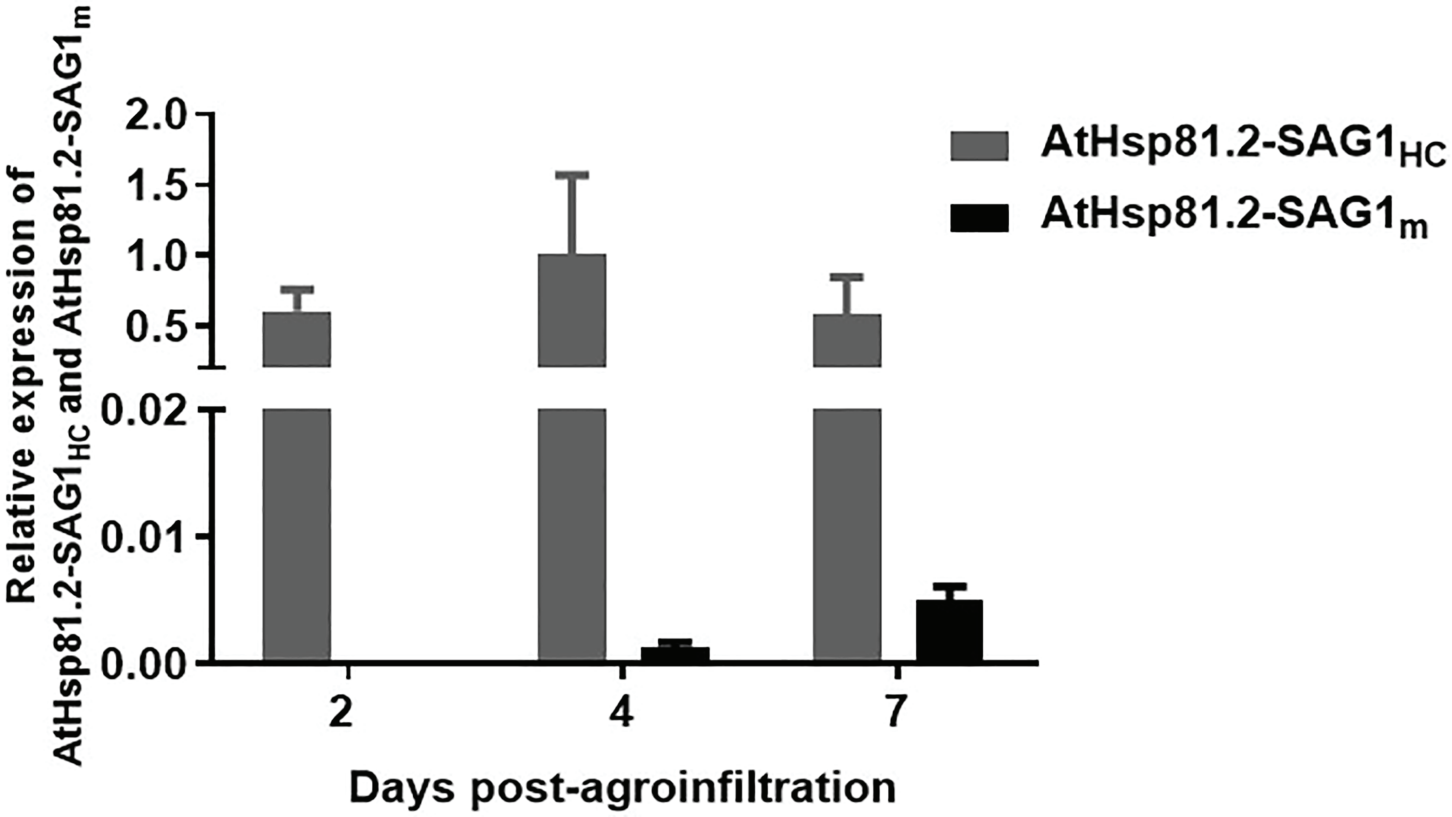
Quantitative real-time polymerase chain reaction (RT-qPCR) analysis of AtHsp81.2–SAG1_HC_ and AtHsp81.2–SAG1_m_ genes in agroinfiltrated leaves. For each time point after agroinfiltration (2, 4, and 7days), relative levels of expression for both genes are shown relative to the *Nicotiana tabacum* elongation factor-alpha (*NtEFα*) gene and are presented as means ± standard error of the mean (SEM) of three independent agroinfiltration assays.

To understand whether differences in protein expression between AtHsp81.2–SAG1_HC_ and AtHsp81.2–SAG1_m_ were a result of differences in mRNA expression, we performed a RT-qPCR analysis. Different expression patterns were observed for each construct (Figure 4); while the AtHsp81.2–SAG1_HC_ mRNA was strongly expressed, with a maximum expression at 4days post-agroinfiltration, the AtHsp81.2–SAG1_m_ mRNA was almost undetectable (~200-fold less than AtHsp81.2-SAG1_HC_) at any of the time points analyzed.

**Figure 5:**
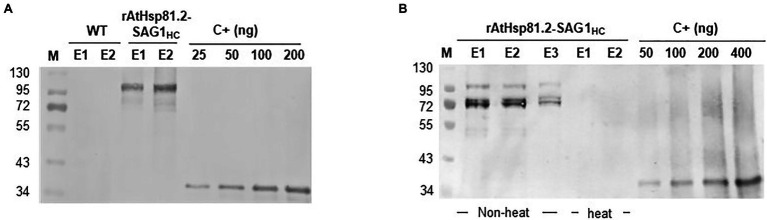
Purification of plant-produced AtHsp81.2–SAG1_HC_ (rAtHsp81.2–SAG1_HC_). **(A)** Western blot analysis of the fraction eluted with 250mm imidazole using anti-rEcSAG1_m_ polyclonal antibody. rAtHsp81.2–SAG1_HC_ (E1 and E2), elutes from *Nicotiana benthamiana* expressing AtHsp81.2–SAG1_HC_; WT (E1 and E2), elutes from non-infiltrated *N. benthamiana*. **(B)** Effect of heat incubation on the rAtHsp81.2–SAG1_HC_ purification procedure. rAtHsp81.2–SAG1_HC_ (E1, E2 and E3), elutes from *Nicotiana benthamiana* expressing AtHsp81.2–SAG1_HC_ without heat incubation; rAtHsp81.2–SAG1_HC_ (E1, and E2), elutes from *Nicotiana benthamiana* expressing AtHsp81.2–SAG1_HC_ after heat incubation at 65°C. Western blot analysis of the fraction eluted with 250mm imidazole was carried out using an anti-rEcSAG1_m_ polyclonal antibody. The protein extractions were heat incubated at 65°C during homogenization, before the purification procedure. Lanes were loaded at equal volumes (10μl) for direct comparison.

AtHsp81.2–SAG1_HC_ was purified using a using a Ni-NTA agarose column. The rAtHsp81.2-SAG1_HC_ was analyzed using Western blot (Figure 5). The 110kDa rAtHsp81.2-SAG1_HC_ (Figure 5A) and degradation products at the height of ~95 and ~72kDa were revealed (Figure 5B). The mean yield of rAtHsp81.2–SAG1_HC_ from independent agroinfiltrations was 60μg/g of FW. As AtHsp81.2 is a member of the family of molecular chaperones that is tolerant to heat stress, we tested whether a heat incubation step at 65°C during homogenization could improve rAtHsp81.2–SAG1_HC_ purification by removing plant host cell proteins (Beiss et al., 2015). However, the rAtHsp81.2–SAG1_HC_ fusion protein was not detected after elution, indicating that either the recombinant protein suffered a conformational change that rendered the His-tag unable to interact with the Ni-NTA agarose column or it was degraded during the purification process (Figure 5B).

### Immunogenicity of Plant Extracts Containing AtHsp81.2-SAG1_HC_ and Purified AtHsp81.2-SAG1_HC_

**Figure 6:**
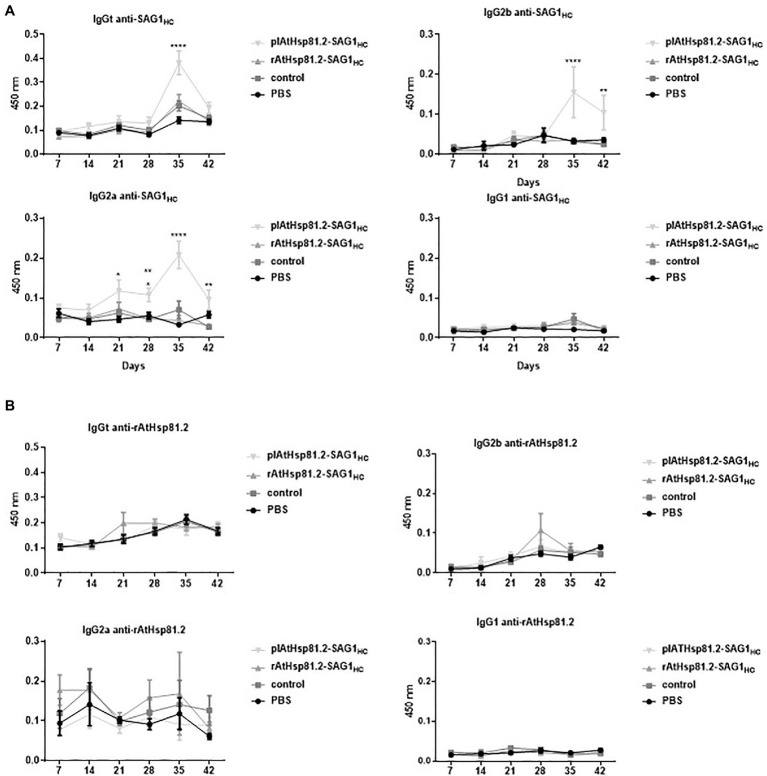
Humoral immune responses against SAG1_HC_ and AtHsp81.2 prior to challenge with *Toxoplasma gondii* cysts in mice. **(A)** Specific immunoglobulin (Ig)Gt, IgG1, IgG2a, and IgG2b antibody production against SAG1_HC_. **(B)** Specific IgGt, IgG1, IgG2a, and IgG2b antibody production against rAtHsp81.2. Serum samples from each group of eight mice were obtained at each time point (7, 14, 21, 28, 35, and 42days post-infection), and serial dilutions were analyzed by direct ELISA. Mouse serum samples were diluted 1:100 to determine specific antibodies (no saturating dilutions). plAtHsp81.2-SAG1_HC_: mice vaccinated with AtHsp81.2-SAG1_HC_-infiltrated leaf extracts, rAtHsp81.2-SAG1_HC_: mice vaccinated with rAtHsp81.2–SAG1_HC_ purified from AtHsp81.2-SAG1_HC_-infiltrated leaves, control: mice vaccinated with non-infiltrated leaf extracts, PBS: mice vaccinated with phosphate-buffered saline 1X. Results are expressed as the mean of the optical density (OD)_630_±standard error of the mean (SEM) and represent two experiments. Statistical analysis was performed by two-way ANOVA using Dunnett’s multiple comparisons test. ^*^p<0.05; ^**^p <0.01; ^****^p<0.0001.

The kinetics of specific antibody production triggered by oral vaccination with AtHsp81.2–SAG1_HC_-infiltrated fresh leaves (plAtHsp81.2–SAG1_HC_ group) or rAtHsp81.2–SAG1_HC_ purified from infiltrated leaves (rAtHsp81.2–SAG1_HC_ group) was determined by serological analysis in C57BL/6 mice. The presence of anti-SAG1_HC_ immunoglobulin (Ig)G antibodies was evaluated over a period of 42days against rEcSAG1m by ELISA (Figure 6). On day 35, mice immunized with plAtHsp81.2–SAG1_HC_ had the highest antibody response, with titers of 12,800 compared with the other groups (Supplementary Figure S2A). Titers remained high until 42days post-immunization (titers of 1,600, Supplementary Figure S2B). In contrast, no anti-SAG1_HC_ IgG was detected in the rAtHsp81.2–SAG1_HC_, control, or PBS groups (Figure 6A, Supplementary Figure S2).

It is generally accepted that a predominant secretion of IgG2a and/or IgG2b over the IgG1 isotype is associated with a Th1-biased immune response, and this is necessary to generate a protection against *T. gondii* (Mévélec et al., 2005). To investigate the immune profile induced after immunization, we evaluated the presence of IgG2a, IgG2b, and IgG1 antibodies in serum samples. Antibody response profiles were different from the two immunized groups: the plAtHsp81.2–SAG1_HC_ group had higher anti-SAG_HC_-specific IgG2a/IgG2b antibody levels from 35days after the first immunization than the rAtHsp81.2–SAG1_HC_ group (titers of 12,800 vs. titers of 100; Figure 6A, Supplementary Figure S2A). On day 42, the anti-SAG1_HC_ IgG2a/IgG2b-specific responses remained higher in plAtHsp81.2–SAG1_HC_ group than in other groups (titers of 6,400 vs. titers of 100; Figure 6A, Supplementary Figure S2B). These analyses demonstrate that mice immunized with the fusion protein maintain high IgG2a/IgG2b levels up to day 42, whereas in the other groups, these antibodies subclasses remain significantly lower following immunization (Figure 6A, Supplementary Figure S2). However, the levels of anti-SAG1_HC_ IgG1 elicited by immunization with plAtHsp81.2–SAG1_HC_ were not significantly higher than those observed in the other groups (Figure 6A, Supplementary Figure S2). These results suggest that immunization with plAtHsp81.2–SAG1_HC_ clearly shows a humoral immune response biased toward T helper 1 (Th1), associated with the production of IgG2a/IgG2b (Figure 6A, Supplementary Figure S2). Furthermore, no rEcAtHsp81.2-specific IgG was detected in any of the immunized mice, indicating undetectable development of humoral immune responses against AtHsp81.2 (Figure 6B).

**Figure 7:**
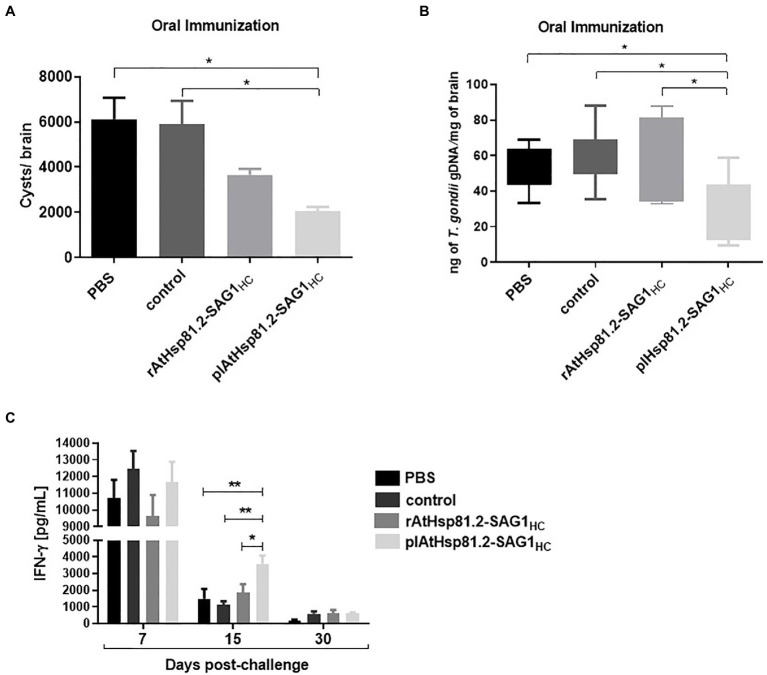
Analysis in orally immunized mice after *Toxoplasma gondii* infection. **(A)** Cyst number per brain. **(B)** Cerebral parasite burden assessed by quantitative PCR (qPCR). Results are expressed as ng parasite genomic DNA per mg of brain tissue. **(C)** Values of interferon (IFN)-γ in serum samples (five mice per group) at 7, 15 (acute infection), and 30 (chronic infection) days after mice were challenged with the cysts. Serum samples were diluted 1:20, and IFN-γ was measured using ELISA. plAtHsp81.2-SAG1_HC_: mice vaccinated with AtHsp81.2-SAG1_HC_-infiltrated leaf extracts, rAtHsp81.2-SAG1_HC_: mice vaccinated with rAtHsp81.2–SAG1HC purified from AtHsp81.2-SAG1_HC_-infiltrated leaves, control: mice vaccinated with non-infiltrated leaf extracts, PBS: mice vaccinated with phosphate-buffered saline 1X. Two weeks after the last boost, mice were challenged by gavages with 20 cysts of the Me49 strain. Each bar represents the group mean±standard error of the mean (SEM). One-way ANOVA was performed using Holm-Sidak’s multiple comparisons test and Tukey’s multiple comparisons test. ^*^p<0.05; ^**^p<0.01.

To determine the immunoprotective effect of the vaccine formulations, mice were challenged with a non-lethal dose of *T. gondii* ME49 cysts 2weeks after the last immunization, and the brain parasite burden was assessed 1month after challenge. All mice survived for 30days. The behavior of mice from plAtHsp81.2–SAG1_HC_ group appeared normal, whereas mice from the other groups exhibited lack of movement and clinical signs of illness, such as diminished exploratory activity, piloerection, hunched appearance, and less grooming behavior. The plAtHsp81.2–SAG1_HC_ group had a significant reduction in cyst burden (by 65%) compared with both control and PBS groups (Figure 7A). Although the rAtHsp81.2–SAG1_HC_ group had a partial reduction in the cyst burden (by 38%) compared with control and PBS groups, it was not statistically significant. Because tissue cysts can vary greatly in size (Dubey et al., 1998), we performed a qPCR to obtain a more accurate indication of parasite burden in mouse brains. As shown in Figure 7B, *T. gondii* was detected in mice from all groups given the cysts; however, once again the plHsp81.2–SAG1 group was the only one that showed significantly lower parasite loads (~20-fold less) in brain tissue compared with those of the remaining groups. In addition, the progression of *T. gondii* infection was assessed using analysis of IFN-γ levels in serum samples from immunized mice that were challenged with cysts. IFN-γ levels remained high in all groups in the first 7days post-challenge; however, significantly higher IFN-γ levels persisted only in the plHsp81.2–SAG1_HC_ group during the acute infection phase (up to 15days post-challenge; Figure 7C). As expected, IFN-γ levels decreased dramatically in all groups 15days after the challenge: ~3,500pg/ml for plAtHsp81.2-SAG1_HC_ vs. ~1,400, ~1,100, and ~1,800pg/ml for PBS, control, and rAtHsp81.2-SAG1_HC_ groups, respectively, which is consistent with the establishment of the chronic infection phase and parasitic cyst formation (Figure 7C). At 7 and 30days after the challenge, IFN-γ levels were not significant between groups (Figure 7C).

**Figure 8:**
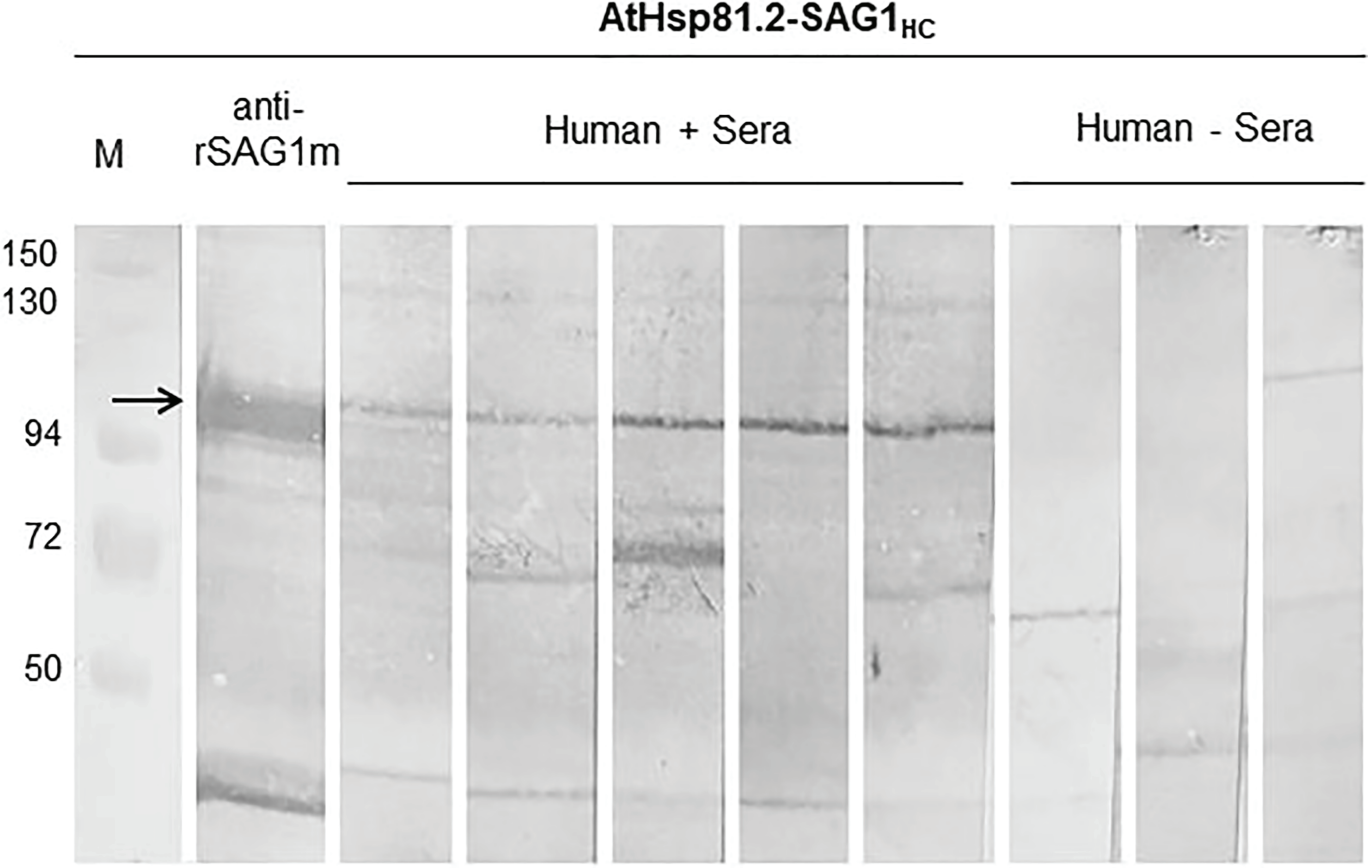
The reactivity of plant-expressed AtHsp81.2-SAG1_HC_. All seropositive samples reacted with a specific band that correspond to the expected size of AtHsp81.2–SAG1_HC_ (110kDa), and it is indicated with arrow. Seronegative human serum samples did not react with any bands of approximately 110kDa. In IgG, the immunoblot assays, leaf extract samples were separated using 10% sodium dodecyl sulfate-polyacrylamide gel electrophoresis (SDS-PAGE) under reducing conditions. Immunoblot profiles of AtHsp81.2–SAG1_HC_ leaf extracts were probed with human negative sera (1:100), human *Toxoplasma*-positive sera (1:100), and mouse anti-rEcSAG1_m_ polyclonal antibody (1:500).

Finally, we used Western blot to assess whether AtHsp81.2–SAG1_HC_-infiltrated fresh leaves be recognized by antibodies in human serum samples from individuals with toxoplasmosis. Our results showed that the antibodies present in all seropositive samples reacted with a specific band (110kDa) with a migration rate corresponding to the expected size of AtHsp81.2–SAG1_HC_ (Figure 8). It is also important to note that seronegative human serum samples did not react with any bands of approximately 110kDa (Figure 7). It should be noted that the human sera did not detect any degradation products of AtHsp81.2-SAG1_HC_. Since the degradation products were purified from the N-terminus, this suggests that the degradation occurs from the C-terminus of SAG1_HC_; and perhaps this region may be relevant for the antibodies present in human sera.

## Discussion

In this study, we demonstrated for the first time to our knowledge that a plant HSP90–SAG1_HC_ fusion protein is expressed as intact protein at levels up to 90μg/g of FW when transiently co-expressed together with suppressor p19 in *N. benthamiana* and that oral vaccination with plant extracts containing AtHsp81.2-SAG1_HC_ in mice elicits appropriate immune responses to prevent toxoplasmosis infection, reducing parasite cyst burden in brain tissue and symptoms of infection. In addition, preliminary results indicate that antibodies in human serum could react with this fusion protein and therefore that this may be a viable system for vaccine production against toxoplasmosis.

In contrast to SAG1_HC_ that contains only the B- and T-cell epitopes, the mature full-length SAG1_m_ protein and mRNA levels were undetectable in AtHsp81.2–SAG1_m_-infiltrated leaves under any of the conditions tested. However, this cannot be associated with a malfunction of the suppressors since they act at the mRNA level (Silhavy and Burgyán, 2004). Our results would support the idea that the lack of AtHsp81.2–SAG1_m_ expression could be attributed to issues at transcription level, either affecting correct mRNA transcription and/or transcription rate. We cannot rule out that some alteration has occurred at the promoter level in the cloning process of AtHsp81.2–SAG1_m_ DNA, which would be a rare event since it is produced by recombination. There is evidence that similar alterations have occurred in a heterologous gene-dependent manner due to some factors that have not been identified yet (Mellor et al., 1985; MacDonald et al., 2017). Recombinant SAG1_m_ could be expressed in plants using viral amplicons (Clemente et al., 2005; Laguía-Becher et al., 2010) or expression vectors that target the protein at the endoplasmic reticulum or the apoplast (Laguía-Becher et al., 2010), suggesting that the nucleotidic sequence encoding SAG1_m_ used in the present study should not be implicated in the lack of expression of this protein. However, other factors related to the mature *SAG1* gene when fusing to the *AtHsp81.2* gene could be interfering, since the mature version of the *SAG1* gene is 429bp longer than encoding SAG1_HC_ version. It is important to mention that neither the AtHsp81.2-SAG1_m_ nor the AtHsp81.2-SAG1_HC_ proteins in *N. tabacum* X-27-8 plants could be detected. In these cases, mRNA levels were not determined, so it remains unclear whether this is due to a malfunction of transcription or translation processes.

Several reports demonstrated that the use of suppressors enhances recombinant protein expression levels and in some cases reaching an accumulation peak at around 6 or 7days post-infiltration (Voinnet et al., 2003; Dhillon et al., 2009; Bashandy et al., 2015; Matsuo et al., 2016; Norkunas et al., 2018). Dhillon et al. (2009) observed that when HC-Pro and p19 are co-infiltrated gave a delayed peak and a long extension of GFP expression. However, GFP expression values were consistently lower for HC-Pro co-agroinfiltration (Dhillon et al., 2009). Similarly, Norkunas et al. (2018) showed that co-expression of p19 resulted in 2.5–4.0-fold increase in recombinant protein expression, while the *Papaya ringspot virus* HC-Pro had no major effect. Our results also suggest that the type of suppressor to be used should be analyzed for each recombinant protein.

Previous studies also observed that concentrations of the *A. tumefaciens* carrying the construct strongly influenced the yields of recombinant protein (Coffman et al., 2010; Kanagarajan et al., 2012; Love et al., 2012; Mardanova et al., 2015; Robert et al., 2016). Garabagi et al. (2012) recommended using *A. tumefaciens* cultures with an OD_600nm_ of 0.2 for binary vectors and cultures with an OD_600nm_ of 0.002 for viral replicons. In contrast, other works have observed that the optimal *A. tumefaciens* concentration to achieve the best yields is an OD_600nm_ of 1.0 (Shah et al., 2013). Besides, Buyel and Fischer (2012) suggested that *A. tumefaciens* solutions with an OD_600nm_ of 0.25 could be used for infiltration without a significant loss of yield compared to those obtained with an OD_600nm_ of 1.0. On the other hand, Wydro et al. (2006) did not show any differences in the levels of transient *GFP* gene expression for the different densities of *A. tumefaciens* suspensions used for infiltration. However, they observed an increase in the expression level of GFP when a mixture of *A. tumefaciens* strains encoding either the *GFP* or *HC-Pro* genes at an OD_600nm_ of 1.0 (1:1 ratio) was used for transient transformation (Wydro et al., 2006). Our results also showed that the yield of recombinant protein depended on the OD_600nm_ of the injected *A. tumefaciens* culture, with the best yield of AtHap81.2–SAG1_HC_ achieved when both AtHsp81.2-SAG1_HC_ and p19 cultures of *A. tumefaciens* were used at an OD_600nm_ of 0.6 (1:1 ratio).

Other authors also noted that recombinant protein expression is dependent on the plant age (Wroblewski et al., 2005; Sheludko et al., 2007; Buyel and Fischer, 2012). Buyel and Fischer (2012) observed that young plants expressed large amounts of the recombinant protein in all leaves compared to older plants. Similarly, Sheludko et al. (2007) found that the level of GFP accumulation depended on *N. benthamiana* developmental stage, increasing in 4- and 6-week-old plants and diminishing in 8- and 10-week-old plants. We observed that the plant age affected the expression yields of AtHsp81.2–SAG1_HC_ in *N. benthamiana*. The highest accumulation levels of AtHsp81.2-SAG1_HC_ were detected in 6-week-old *N. benthamiana* plants compared to 8-week-old *N. benthamiana* plants. Taken together, our findings are consistent with those obtained by other authors, suggesting that highest accumulation levels of the recombinant proteins would be achieved during the vegetative growth of the plants.

Finally, several studies have examined the effect of light intensity during the post-agroinfiltration process on recombinant protein content (Zambre et al., 2003; Cazzonelli and Velten, 2006; Fujiuchi et al., 2016), and in some of them, the content of recombinant protein was associated with temperature changes (Buyel and Fischer, 2012, 2013; Fujiuchi et al., 2016). Van Iersel (2017) reported that LED lights have better control of temperature and humidity, two parameters of high impact on the production of recombinant proteins. Likewise, LED lights contribute to accelerating the germination time and increasing the plant biomass (Holtz et al., 2015). We also observed that light environment control is important for recombinant protein content. AtHsp81.2–SAG1_HC_ yield was improved using LED lighting. Therefore, the results obtained here could be explained by the best control of these two parameters due to using LED lights. Therefore, LED lights could also have a positive impact on the achieved yields of the recombinant protein.

Here, the maximum AtHsp81.2–SAG1_HC_ expression levels yielded up to 90μg/g FW. Previously, the transient transformation of SAG1 was evaluated using a vacuum infiltration system (Clemente et al., 2005; Laguía-Becher et al., 2010). Initially, Clemente et al. (2005) determined an expression range for cytosolic SAG1_m_ of between 6 and 10μg/g of FW, which varied depending on different viral promoters of transient expression. In that study, the expression system was based on a potato virus X amplicon and the SAG1 sequence was driven by either the triple gene block open reading frame 1 (ORF 1) or the viral coat protein subgenomic promoters. Later, Laguía-Becher et al. (2010) achieved an expression level of 1.3μg/g of FW when SAG1_m_ was fused to an endoplasmic reticulum-targeting signal peptide under a CaMV 35S promotor with double enhancer element. More recently, Albarracín et al. (2015) investigated the chloroplast transformation technology as a method to improve the SAG1_m_ expression. However, the expression levels of chloroplast SAG1_m_ were lower (from 10- to 100-fold less) to those obtained by the previous studies (Clemente et al., 2005; Laguía-Becher et al., 2010). In fact, results obtained by Albarracín et al. (2015) showed that SAG1_m_ expression levels were significantly increased (to 100μg/g of FW, by up to 500-fold) when the antigen of interest was fused to LiHsp83 from the protozoan pathogen *Leishmania infantum* (LiHsp83) respect to SAG1_m_ in transplastomic *Nicotiana tabacum* cv. Petite Havana. Therefore, the authors concluded that the use of LiHsp83 as carriers improves the expression yield of recombinant proteins (Albarracín et al., 2015). Interestingly, cytosolic AtHsp81.2–SAG1_HC_ accumulation obtained from agroinfiltrated *N. benthamiana* leaves were similar to those obtained for LiHsp83–SAG1_m_ expressed in chloroplasts. This suggests that agroinfiltration in tobacco plants could produce high yields of recombinant proteins without the need to produce transgenic plants, simplifying the method of production and the use of HSP90 as a carrier for recombinant proteins could be implemented in plant-based expression systems.

Our humoral response analysis demonstrated that oral immunization with plAtHsp81.2–SAG1_HC_ induced the production of high levels of anti-SAG1_HC_ antibodies in mice, suggesting that the B-cell antigenic epitopes contained in the C-terminal region of SAG1 are sufficient to elicit a strong anti-SAG1_HC_ response. Furthermore, serum samples from plAtHsp81.2–SAG1_HC_-immunized mice contained significantly higher levels of IgG2a and IgG2b anti-SAG1_HC_ antibodies compared with both control and PBS groups. In general, an immune response that induces the production of IgG2a and/or IgG2b antibodies is mainly mediated by Th1 cells, whereas the production of IgG1 antibodies is mediated by Th2 cells (Snapper and Paul, 1987; Snapper et al., 1988; Stevens et al., 1988). Several reports showed that adjuvant-free recombinant SAG1 used in mouse immunizations does not enhance an effective humoral response against *T. gondii* infection or enhance a non-protective Th2 antibody response (Naeem et al., 2018; Abasi et al., 2019; Sánchez-López et al., 2019; Pagheh et al., 2020). In fact, SAG1 immunization was always analyzed with an adjuvant to generate a strong Th1 immune response (Wang and Yin, 2014). Previous studies evaluating the adjuvant capacity of the murine heat shock protein Gp96 fused to antigenic peptides from different pathogenic viruses showed that Gp96 significantly increases the specific IgG2a/b response against the carried peptides (Chen et al., 2013; Ju et al., 2014; Niu et al., 2016). In addition, some authors showed that while a high immune response occurs against the fused antigen of interest, they did not observe an immune response against Gp96 or HSP90. In fact, they observed that after immunization with HSP90-antigen fusion protein, Gp96/HSP90 alone failed to stimulate the production of IFN-γ or cytotoxic T-lymphocyte response (Rapp and Kaufmann, 2004; Yan et al., 2007; Mohit et al., 2012; Chen et al., 2013; Corigliano et al., 2013). Similar results were observed in our laboratory using recombinant *E. coli*-purified NbHsp90.3 as a fusion strategy (Corigliano et al., 2013; Sánchez-López et al., 2019). Here, no development of humoral immune responses against AtHsp81.2 was observed since no rAtHsp81.2-specific IgG was detected in any of the immunized mice. We hypothesize that HSP90s contribute to the carrier/adjuvant function, as adjuvants presenting the antigenic peptides into the antigen cross-presentation pathway. However, more studies are needed to further elucidate this issue.

Previously, Laguía-Becher et al. (2010) showed that a heterologous protocol based on oral immunization with plant-made SAG1_m_ followed by a rEcSAG1_m_ intradermal boost was sufficient to elicit a significant level of protection (50%). Other results have shown a promising protective effect in models of *T. gondii* infection using an oral immunization protocol containing LiHsp83–SAG1_m_, as mice immunized with this protein showed a significant decrease in parasite load (60%) compared with a control group (Albarracín et al., 2015). In comparison, plAtHsp81.2–SAG1_HC_-immunized mice had a significant reduction in the number of cysts per brain as well as lower parasite loads in brain tissue, achieving 65% protection compared with control and PBS groups without the use of a boost strategy. Taken together, our results suggest that the oral immunization with AtHsp81.2–SAG1_HC_ expressed in plant leaf extracts enhances the immunogenicity without generating associated pathogenic effects.

Recombinant hepatitis B surface antigen (HBsAg) was one of the first antigens produced in plants (Mason et al., 1992) and used in oral immunizations (Kong et al., 2001). The authors observed that oral immunization with purified yeast-derived HBsAg did not induce an immune response, while oral immunization with transgenic HBsAg potatoes stimulated an effective one. Based on these results, the authors suggested that natural encapsulation of the antigen may protect it from degradation in the digestive tract and that the antigen could be released near an immune effector site in the gut. Later, several reports demonstrated that plant tissue might prolong the residence time on the mucosa, thereby increasing antigen uptake and enhancing the immune response (Hickey and Garmise, 2009; Velasquez et al., 2011; Arntzen, 2015; Merlin et al., 2017). It is worth mentioning that there was no significant reduction in the parasite load of rAtHsp81.2–SAG1_HC_-immunized mice compared with the control and PBS groups, especially when the load of *T. gondii* genomic DNA was analyzed in brains of challenged mice, supporting the results that suggest that only a minimal or none immune response has been generated. Similar to that observed by other authors, this could happen because purified protein is sensitive to digestive tract degradation. This indicates that the antigen-containing plant extract could be used as a vehicle for delivery in oral immunizations, potentially avoiding its degradation during passage through the intestinal tract.

Because *T. gondii* has been shown to elicit a strong Th1 immune response (Yap and Sher, 1999), we analyzed the cytokine profile in mouse serum after being given *T. gondii* cysts. As expected, after being challenged with the cysts, high levels of IFN-γ were observed in serum samples from all groups of immunized mice during the acute phase of infection. However, the IFN-γ serum levels remained elevated up to 15days post-challenge in the plAtHsp81.2–SAG1_HC_-immunized group alone. The higher levels of IFN-γ in this group could be related to the higher levels of protection observed in this group. Some studies have suggested that the Th1 response induced after vaccination could result in high lethality in the C57BL/6 mouse strain that we used in our study (Vercammen et al., 2000; Liesenfeld, 2002; Martin et al., 2004; Rachinel et al., 2004); however, in our study, the modulation of the Th1 response did not affect the survival rate of plAtHsp81.2–SAG1_HC_-immunized mice. Finally, SAG1_HC_ was recognized by antibodies present in serum samples from people with toxoplasmosis, indicating that plant-expressed AtHsp81.2-SAG1_HC_ maintains its structural integrity. These results suggested that, when fused with AtHsp81.2, SAG1_HC_ could retain the capacity to elicit an immune response if used in a vaccine. This also demonstrates SAG1_HC_ expressed in plant tissue has enough epitopes to react with antibodies from people with toxoplasmosis.

## Conclusion

Our study demonstrated that plant HSP90, a chaperone of non-pathogenic origin, exhibits adjuvant properties when fused to the *T. gondii* SAG1 antigen and transiently expressed in plants. Plant extract containing AtHsp81.2-SAG1_HC_ was capable of inducing a Th1-biased humoral protective immune response against *Toxoplasma* infection suggesting that it could be used as a vehicle for delivery in oral immunizations. In addition, plant HSP90 can also function as a carrier of antigenic proteins such as SAG1_HC_, which contains key antigenic epitopes of the full SAG1 protein. We identified optimal conditions for the expression of this particular protein based on a range of infiltration and plant growth parameters. The optimal conditions included the use of p19 suppressor, 6-week-old plants, 7-day time of harvest, *A. tumefaciens* cultures with an OD_600nm_ of 0.6 for binary vectors and LED lighting. Hence, plant-based expression system is a suitable and powerful biotechnological platform for immunogenic antigen production against toxoplasmosis.

## Data Availability Statement

The original contributions presented in the study are included in the article/Supplementary Material, further inquiries can be directed to the corresponding author.

## Author Contributions

ES-L participated in the design of the study, made the constructs, carried out the agroinfiltration experiments, analyzed all the recombinant protein expression level data, and carried out the immunological studies. MC supervised and helped to carried out the immunological studies and to draft the manuscript. SO carried out the qRT-PCR experiments. VR-D helped to carry out agroinfiltration experiments. LM-M helped to carry out the immunological studies. MR helped to carry out the Western blots with human serum samples. VS helped to analyze data and to edit the manuscript. SA collected the human serum samples, helped to design experiments, and edited the manuscript. MC designed the experiments, wrote the manuscript, and supervised the project. All authors read and approved the final manuscript.

## Funding

This work was supported by PICT 2016-0310, PICT 2016-0621, PICT 2016-0113 of the Agencia Nacional de Promoción Científica y Tecnológica (ANPCyT, Argentina) and R01AI129807 (NIAID, NIH, to SA). The study also received institutional support from the Universidad Nacional de General San Martín (UNSAM, Argentina).

## Conflict of Interest

The authors declare that the research was conducted in the absence of any commercial or financial relationships that could be construed as a potential conflict of interest.

## Publisher’s Note

All claims expressed in this article are solely those of the authors and do not necessarily represent those of their affiliated organizations, or those of the publisher, the editors and the reviewers. Any product that may be evaluated in this article, or claim that may be made by its manufacturer, is not guaranteed or endorsed by the publisher.

## Footnotes

^1^ https://www.cdc.gov/parasites/toxoplasmosis/index.html

## Supplementary Material

Supplementary Figure S1 The nucleotide and amino acid sequence of the native SAG1 from *T. gondii*. **(A)** The signal peptide and the C-terminal hydrophobic region to generate the mature SAG1_77-322_ version are indicated in grey letters. **(B)** Arrows indicate the cleavage sites to generate the mature SAG1 version (SAG1_m_). The underlined sequence corresponds to the truncated version of the SAG1 protein called SAG1_HC_. The bold letters and bold and italics letters indicate the B- and T-cell response epitopes sequences, respectively.

Supplementary Figure S2 SAG1_HC_-specific immunoglobulin (Ig)Gt, IgG1, and IgG2b antibody titers of serum samples. At 35 and 42days after immunization, blood samples from the eight mice in each group were obtained. Serum samples from each group of mice were pooled, and serial dilutions were analyzed by direct ELISA. plAtHsp81.2-SAG1_HC_: mice vaccinated with AtHsp81.2-SAG1_HC_-infiltrated leaf extracts, rAtHsp81.2-SAG1_HC_: mice vaccinated with rAtHsp81.2–SAG1_HC_ purified from AtHsp81.2-SAG1_HC_-infiltrated leaves, control: mice vaccinated with non-infiltrated leaf extracts, and PBS: mice vaccinated with phosphate-buffered saline 1X. Results are expressed as endpoint titers, defined as the highest dilution that gave a value above the cutoff. Cutoff values are those of the control and PBS groups.
