# Supplementary figures and images for: Oral Immunization With a Plant HSP90-SAG1 Fusion Protein Produced in Tobacco Elicits Strong Immune Responses and Reduces Cyst Number and Clinical Signs of Toxoplasmosis in Mice
Source: Front Plant Sci. 2021 Oct 4;12:726910. doi: 10.3389/fpls.2021.726910 (PMC8525317; doi:10.3389/fpls.2021.726910)

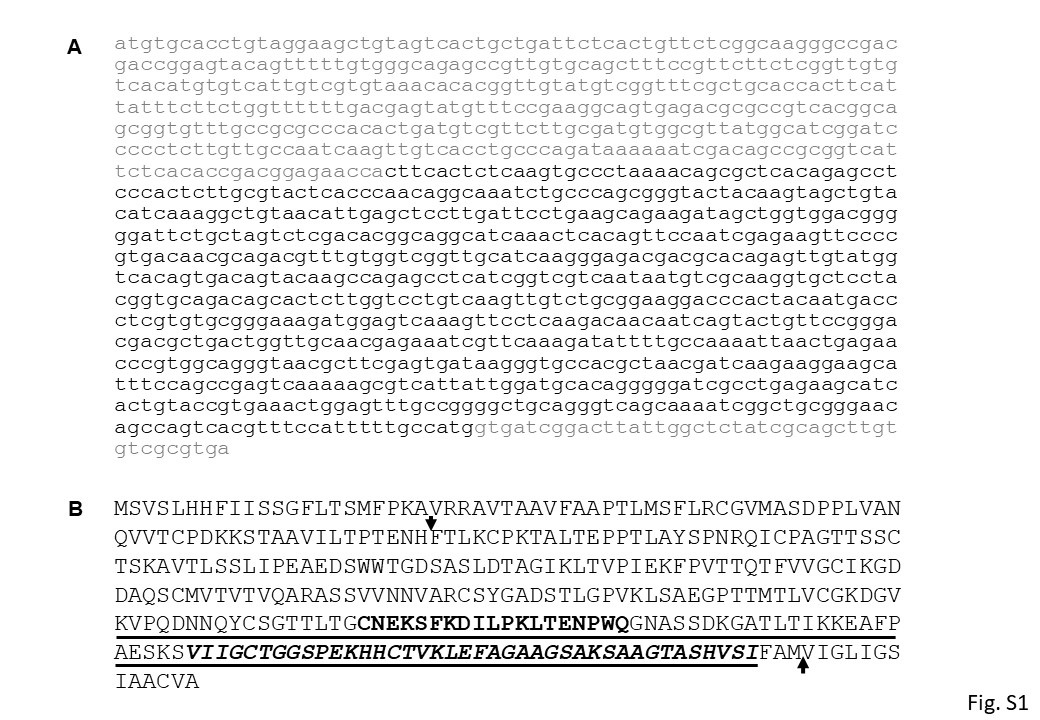

Supplement: Supplementary Figure S1 — The nucleotide and amino acid sequence of the native SAG1 from T. gondii. (A) The signal peptide and the C-terminal hydrophobic region to generate the mature SAG177-322 version are indicated in grey letters. (B) Arrows indicate the cleavage sites to generate the mature SAG1 version (SAG1m). The underlined sequence corresponds to the truncated version of the SAG1 protein called SAG1HC. The bold letters and bold and italics letters indicate the B- and T-cell response epitopes sequences, respectively. [file Image_1.JPEG]
